# A graphical user interface for an electron monitor unit calculator using a sector‐integration algorithm and exponential curve‐fitting method

**DOI:** 10.1120/jacmp.v7i1.2183

**Published:** 2006-02-21

**Authors:** James C.L. Chow, Grigor N. Grigorov, Christopher MacGregor

**Affiliations:** ^1^ Medical Physics Department Grand River Regional Cancer Center Grand River Hospital P.O. Box 9056, 835 King Street, West Kitchener Ontario N2G 1G3; ^2^ Department of Physics University of Waterloo 200 University Avenue Waterloo Ontario N2L 3G1 Canada

**Keywords:** electron radiotherapy, monitor unit, output factor, graphical user interface, computer programming

## Abstract

A new electron monitor unit (MU) calculator program called “eMUc” was developed to provide a convenient electron MU calculation platform for the physics and radiotherapy staff in electron radiotherapy. The program was written using the Microsoft Visual Basic.net framework and has a user‐friendly front‐end window with the following features: (1) Apart from using the well‐known polynomial curve‐fitting method for the interpolation and extrapolation of relative output factors (ROFs), an exponential curve‐fitting method was used to obtain better results. (2) A new algorithm was used to acquire the radius in each angular segment in the irregular electron field during the sector integration. (3) A comprehensive graphical user interface running on the Microsoft Windows operating system was used. (4) Importing irregular electron cutout field images to the calculator program was simplified by using only a commercial optical scanner. (5) Interlocks were provided when the input patient treatment parameters could not be handled by the calculator database accurately. (6) A patient treatment record could be printed out as an electronic file or hard copy and transferred to the patient database. The data acquisition mainly required ROF measurements using various circular cutouts for all the available electron energies and applicators for our Varian 21 EX linear accelerator. To verify and implement the calculator, the measured results using our specific designed irregular and clinical cutouts were compared to those predicted by the calculator. Both agreed well with an error of ±2%.

PACS number(s): 87.53.Fs; 87.53.Hv; 87.66.‐a

## I. INTRODUCTION

Electron radiotherapy is used to treat superficial lesions and tumors at shallow depths under the skin. However, the dose per monitor unit (MU) is difficult to predict due to the variation in electron scattering with a variety of linear accelerators, beam energies, source‐to‐surface distances (SSDs), and beam collimation systems.^(^ ^1^ ^–^ ^4^ ^)^ As a result, the dose per MU of a specific treatment is usually measured individually in order to achieve an acceptable accuracy. Obviously, this patient‐specific measurement consumes a lot of resources and man‐hours in the physics department. The dose per MU can be calculated by the relative output factor (ROF) for the electron beam. ROF is defined as the ratio of the dose rate in water at a reference point with a custom cutout to the dose rate under the beam calibration condition. The reference point can be either the depth of maximum dose $\left(d_{m}\right)$ or a reference depth $\left(d_{\text{ref}}\right)$ defined by the AAPM TG‐51 protocol.^(5)^ Typically, the LINAC is calibrated at the reference point in water using a $10\times 10\;{\text{cm}}^{2}$ applicator and cutout at SSD=100cm to obtain 1 cGy/MU with an acceptable uncertainty of ±2%.^(6)^ Therefore, determining the ROF for a specific treatment is the first step in calculating the dose per MU for the electron radiotherapy. In this paper, unless mentioned otherwise, all ROFs were measured at SSD=100cm.

To avoid time‐consuming patient‐specific ROF measurements, much work has been done in ROF prediction based on different algorithms such as the Gaussian pencil beam model plus collimator scattering,^(^ ^7^ ^,^ ^8^ ^)^ the lateral buildup ratio,^(9)^ the two‐source model,^(10)^ and sector integration.(11) These algorithms attempt to predict the ROF of an irregular electron field using a database of parameters based on measurements, which were simplified as much as possible. For example, the AAPM TG‐70 protocol suggests using the lateral buildup ratio based on the pencil beam model, which separates the applicator factors from the in‐phantom scatter contribution by measuring the fractional depth dose normalized to the surface. The fractional depth doses are measured as a function of field size for one small field (e.g., 2 cm diameter) and one infinitely large field (e.g., $10\times 10\;{\text{cm}}^{2}$).^(^ ^12^ ^,^ ^13^ ^)^ However, the work of the AAPM TG‐70 protocol is still in progress.

Most of the commercial radiation treatment‐planning systems (RTPSs) can calculate the electron MU based on the above algorithms; however, an in‐house electron MU calculator is desired as a QA tool for the RTPSs, as well as for predicting the MU for an emergency treatment without a plan from the RTPS. The calculator program should be reliable, user‐friendly, accurate, and compatible with the most common personal computer operating system—Microsoft Windows—so that all radiation oncology staff will find it easy to use.

The aim of this paper is to present a clinical in‐house electron MU calculator called “eMUc.” This calculator program uses the sector‐integration algorithm to predict the ROF of an irregular electron field and then calculates the MU. The program has the following features:

1. By using the polynomial curve‐fitting method, or even better, the exponential curve‐fitting method, in the clinical cutout size range (i.e., cutout radius >2cm), the ROF of an irregular field can be accurately predicted with an error of ±2% compared to the measurement.
2. A new algorithm was used to measure the radius in each divided angular segment in the irregular field when doing the sector integration.
3. A graphical user interface approach was used.
4. The image of an irregular cutout field can be converted to a JPEG file or the like by using a commercial optical scanner, and then imported into the calculator program. This avoids using the bulky film digitizer.
5. Interlocks are initiated if the calculator cannot predict the MU accurately according to the scope of the database and the accuracy of the fitting curve.
6. A patient treatment record, including the calculated electron MU, patient information, and all treatment setup parameters, can be converted to an electronic file for the patient database or printed out as a hard copy.

In this paper, the ROF database was acquired using our Varian 21 EX LINAC, which can produce clinical electron energies of 4 MeV, 6 MeV, 9 MeV, 12 MeV, and 16 MeV. Five electron applicators of dimensions $6\times 6\;{\text{cm}}^{2},\;10\times 10\;{\text{cm}}^{2},\;15\times 15\;{\text{cm}}^{2},\;20\times 20\;{\text{cm}}^{2}$, and $25\times 25\;{\text{cm}}^{2}$ were used in the measurement.

## II. METHODS AND MATERIALS

### A. Theory

The electron MU in this paper is calculated using the effective SSD technique^(^ ^14^ ^–^ ^16^ ^)^:

(1) $$\text{MU}=\frac{\text{dose}/\text{fraction}}{D_{0}C\frac{\text{IDL}}{100}\text{ROF}{\left(\frac{{\text{SSD}}_{\text{eff}}+d_{0}}{{\text{SSD}}_{\text{eff}}+d_{0}+g}\right)}^{2}},$$

where $D_{0}$ is the absolute calibrated dose (1cGy/MU) at SSD=100cm and $d_{\text{ref}}$ in water according to the AAPM TG‐51 protocol. The correction factor *C* corrects the difference between the percentage depth doses for $d_{\text{ref}}$ and $d_{m}$, which is the depth used in planning and prescribing the patients in our center. IDL is the isodose line of the prescription. ${\text{SSD}}_{\text{eff}}$ is the effective SSD, $d_{0}$ is the depth of treatment, and *g* is the air gap, which is the difference between the regular SSD of 100 cm and the treatment SSD. In Eq. (1), the dose/fraction, $d_{0}$, and IDL are decided by the oncologist. The values of $D_{0},\;C$, and ${\text{SSD}}_{\text{eff}}$ are predetermined by the physicist for the LINAC with all the available electron energies. The value of *g* is measured by the radiotherapists during the setup and/or simulation of the patient before the treatment. The ROF depends on the shape of the irregular electron field and varies with beam energy and applicator size. It should be noted that for the Varian 21 EX LINAC, the distance between the machine isocenter and the bottom surface of the applicator is 5 cm. When the treatment SSD is equal to 100 cm (i.e., g=0), the inverse square law term (IDL/100) in the denominator of Eq. (1) becomes 1.

For the irregular field, the ROF is calculated using the sector‐integration formula:

(2) $$\text{ROF}=\frac{1}{n}\sum_{i=1}^{n}{\text{ROF}}_{i},$$

where *n* is the number of angular segments divided into an irregular field with angles of 360°/n. ${\text{ROF}}_{i}$ is the ROF for a circular field with the radius equal to the distance measured from the field central axis to the edge in each individual angular segment. This model can be applied to off‐axis points or cutouts with the radius vector crossing a blocked field at the depth of $d_{\text{ref}}$. The part of radius vector crossing the blocked field can be subtracted from the original radius vector at the center. The resulting vector will then be used to calculate the ROF. To calculate the ROF for an irregular field, a database of ROFs for circular fields of varying sizes, electron beam energies, and applicator sizes should be predetermined. In this paper, various circular cutouts were fabricated for all five applicators, and measurements were taken to determine the ROFs for the five electron beam energies. The measured ROFs at the depth of $d_{\text{ref}}$ were then plotted against the cutout radii corresponding to various combinations of energies and applicators, so that ROFs for any other cutout sizes could be obtained through interpolation or extrapolation using curve‐fitting methods and the plots.

For calculating the ROF of a square or rectangular cutout, the following well‐known formula was used^(17)^:

(3) $$\text{ROF}(X,Y)=\sqrt{\text{ROF}(X,X)\times \text{ROF}(Y,Y)},$$

where ROF(*X*, *Y*) is the ROF of a rectangular cutout with side lengths of *X* and *Y*. ROF(*X*, *X*) and ROF(*Y*, *Y*) are ROFs of square cutouts with side lengths of *X* and *Y*, respectively. It should be noted that Eq. (3) can only be used for the situation of constant collimator setting. Applicator factors are needed in other situations.

### B. Computer programming

**Figure 1:**
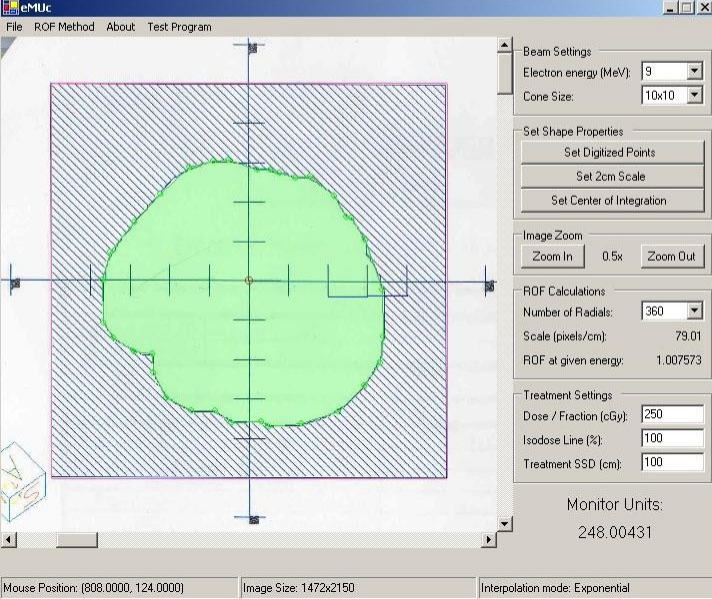
The front‐end window of eMUc showing the imported irregular electron field image in the left‐hand side and the patient treatment parameters in the right‐hand side. The option of a polynomial or exponential curve‐fitting method can be selected through the “ROF Method” pulldown menu in the top of the window.

The Microsoft Visual Basic.net framework version 1.0 was used to develop the electron MU calculator program. The program works on Microsoft Windows and contains a front‐end window, routines for sector integration and MU calculation, an interface for importing cutout images, and a database for measurements and curve fitting. The front‐end window, as shown in Fig. 1, includes all the treatment parameter entries, such as electron beam energy, applicator size, prescribed dose, IDL, and SSD, used in the MU calculation so that the user can input them conveniently into the computer program.

**Figure 2:**
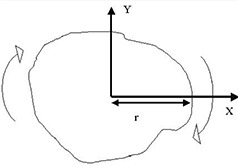
Schematic diagram showing the algorithm to acquire the radius in each divided angular segment in the irregular field. Instead of rotating the vectors from the central axis toward the field edge, the whole field is rotated cutting through the positive *x*‐axis.

For the sector‐integration routine, a new algorithm was used to find the radius of each angular segment in the irregular field: Instead of rotating a vector directed from the field central axis to the static field edge to measure the radius for each angular segment, the irregular field was rotated in the opposite direction, and the positive *x*‐axis was used as a static vector. The distance between the field central axis and the edge along the positive *x*‐axis was measured as radius $r_{i}$ for each angular segment step by step as shown in Fig. 2. This routine continued until the whole cutout field had been rotated 360°, greatly reducing the length of the programming code needed to determine $r_{i}$ compared to the conventional algorithm, which rotates the central axis vector.^(11)^ In addition, since modern computing speed is very fast, the number of angular segments (a user‐selectable option) can be increased up to 360 compared to typically 36 segments or fewer as suggested in previous works.^(^ ^11^ ^,^ ^18^ ^–^ ^20^ ^)^ Increasing the number of angular segments in this manner does not increase the accuracy of the ROF calculation except in cases where the shape is highly irregular.

The integration parameters are stored in a Microsoft Access database, which is loaded during program startup. The database contains three tables: The first table stores the parameters used in Eq. (1), such as $C,\;d_{m},\;d_{\text{ref}}$, and ${\text{SSD}}_{\text{eff}}$, which depend only on the beam energy selected. The second table stores the fitting curve parameters of 25 combinations (5 applicators and 5 energies) for the plotting of ROFs against the radii of circular fields using the exponential and polynomial method. This table was used for predicting the ROF using the interpolation and extrapolation technique. The third table stores a large collection of ROFs for various combinations of square sizes, cone sizes, and electron energies; linear interpolation between points is then performed when calculating the ROF of a square or rectangular field. The database is password‐protected so that only users with authority, such as the treatment‐planning resource physicist, are allowed to change the content.

### C. Data acquisition and measurement

The values of $C,\;d_{m},\;d_{\text{ref}}$, and ${\text{SSD}}_{\text{eff}}$ were measured using a scanning water tank system (RFA 300, Scanditronix Medical AB with Omni Pro 6 software). A waterproof high‐doped p‐type silicon diode (Scanditronix Medical AB, EFD‐3G) was used to measure both the beam profiles and percentage depth doses at the central beam axis. The thickness of the silicon chip is 0.5 mm, and the diameter of the active area is 2 mm. The values of $d_{m}$ and $d_{\text{ref}}$ for 4 MeV, 6 MeV, 9 MeV, 12 MeV, and 16 MeV were measured with a standard $10\times 10\;{\text{cm}}^{2}$ cutout and a $10\times 10\;{\text{cm}}^{2}$ applicator at SSD=100cm. The ${\text{SSD}}_{\text{eff}}$ values for the five energies and applicators were measured according to the procedure in Ref. 15. The values of ${\text{SSD}}_{\text{eff}}$, which vary with the electron field size and beam energy, are shown in Table 1(a), and the values of $d_{m}$ and $d_{\text{ref}}$, which vary with energy for the standard $10\times 10\;{\text{cm}}^{2}$ applicator and cutout, are shown in Table 1(b).

**Table 1:** (a) The effective SSD $\left({\text{SSD}}_{\text{eff}}\right)$ and (b) the depth of maximum dose $\left(d_{m}\right)$ and reference depth $\left(d_{\text{ref}}\right)$ varied with the electron energies for the standard $10\times 10\;{\text{cm}}^{2}$ applicator and cutout.

(a)	Applicator Size (cm)	Field Size (cm)	Electron Beam Energy (MeV)			
			6	9	12	16
	6×6	6×6	68	79	80	81
		5×5	61	75	78	81
		4×4	48	63	71	79
		4×6	56	70	75	80
	10×10	10×10	83	89	87	88
		6×6	76	82	82	82
		5×5	65	74	79	80
		4×10	61	70	76	81
	15×15	15×15	87	92	95	94
		6×6	75	77	82	85
		5×15	71	77	84	87
	20×20	15×15	93	94	94	95
		6×6	73	78	81	89
		5×15	73	80	82	89
	25×25	15×15	94	94	94	92
		6×6	73	81	85	85
		5×15	74	81	85	87

	(b)	Electron energy (MeV)	$d_{m}\;(\text{cm})$	$d_{\text{ref}}\;(\text{cm})$
		4	0.70	0.63
		6	1.40	1.30
		9	2.15	2.06
		12	2.90	2.92
		16	3.20	3.88

To measure the content of the second table for the database in Section II.B, ROFs of various circular cutouts for the five electron energies were measured using the Varian 21 EX LINAC. Circular cutouts with different sizes for the five applicators were fabricated; their radii are shown in Table 2. A scanning water tank and an electron diode positioned perpendicular to the water surface was used to measure the ROFs at SSD=100cm. For small cutouts (radius <2cm), radiographic film (Kodak XV) and solid water phantom slabs were used to verify the measurements. The ROFs for the square cutouts were measured using a similar procedure. All ROFs were measured at the depth of $d_{\text{ref}}$ in this study. Since ROF is an output ratio, the uncertainty contributed by the dosimeter should be very small. The uncertainty in the measurement was mainly contributed by the experimental setup and the positional error of the circular cutout at the center of the applicator frame.

**Table 2:** The list of electron circular cutouts fabricated for each applicator used in measuring the relative output factors

Applicator	Prepared Circular Cutout Radius (mm)							
6cmx6cm	10.0	12.5	15.0	20.0	25.0	30.0		
10cmx10cm	10.0	15.0	20.0	30.0	40.0	50.0		
15cmx15cm	10.0	20.0	30.0	40.0	50.0	60.0	75.0	
20cmx20cm	10.0	20.0	30.0	40.0	50.0	75.0	100.0	
25cmx25cm	10.0	20.0	30.0	40.0	50.0	75.0	100.0	125.0

### D. Data analysis and curve fitting

Using the measured ROFs for various circular cutouts, the ROFs were plotted against the radii of cutouts at each applicator size for specific electron energies. Twenty‐five graphs for all combinations of the five electron beam energies and applicators were therefore plotted. Since each graph only had five to seven data points, it was necessary to interpolate or extrapolate so that ROFs for any electron field could be accurately predicted. In eMUc, two different kinds of curve fitting, polynomial and exponential, were used. The user can select between these two methods in order to carry out the MU calculation according to different clinical situations. The linear polynomial method^(^ ^22^ ^–^^24^^)^ used in the calculator program contained six fitting coefficients $(a_{0},\;a_{1},\;a_{2},\;a_{3},\;a_{4}$, and $a_{5}$):

(4) $$y=a_{0}+a_{1}x+a_{2}x^{2}+a_{3}x^{3}+a_{4}x^{4}+a_{5}x^{5},$$

while the exponential method used the formula

(5) $$y=b_{1}-e^{b_{2}x+b_{3}},$$

and uses three fitting coefficients ($b_{1},\;b_{2}$, and $b_{3}$). MATLAB version 7.0 was used for the curve fitting.

### E. Clinical implementation

**Figure 3:**
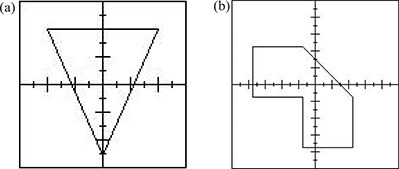
The shapes of the specific irregular cutouts to test the calculator: (a) the shape of the cutout for the $6\times 6\;{\text{cm}}^{2}$ applicator; (b) the shape of the cutout for the $10\times 10\;{\text{cm}}^{2}$. The shapes of other irregular cutouts for $15\times 15\;{\text{cm}}^{2},\;20\times 20\;{\text{cm}}^{2}$, and $25\times 25\;{\text{cm}}^{2}$ applicators are similar to Fig. 3(b).

When the data acquisition and ROF curve fitting were finished and the results were input into the database, the calculator program was ready to be commissioned for clinical use. Specific irregular cutouts were designed to verify the predicted ROFs. Cutouts as shown in Figs. 3(a) and 3(b) were made for the five applicators. In addition, the ROFs of irregular cutouts used for clinical treatments in our center were also compared to the predicted results for verification. The inverse square law using the effective SSD technique for the calculator program was verified with dose measurements at different SSDs. The calculated electron MU from the program using all the input parameters must be verified to make sure that the accuracy is acceptable (i.e., error <±2%) before starting the program clinically.

## III. RESULTS

**Figure 4:**
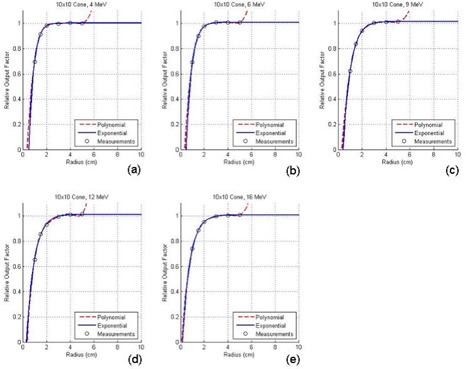
Relative output factors plotted against the radii of the circular cutouts for the $10\times 10\;{\text{cm}}^{2}$ applicator at electron energies of (a) 4 MeV, (b) 6 MeV, (c) 9 MeV, (d) 12 MeV, and (e) 16 MeV.

**Figure 5:**
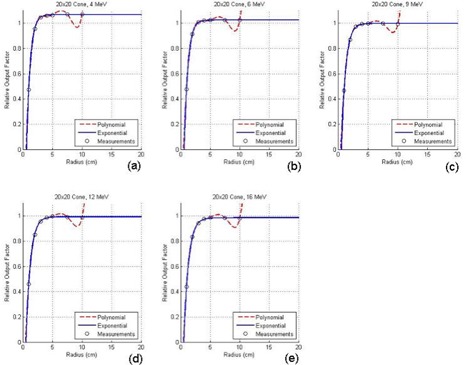
Relative output factors plotted against the radii of the circular cutouts for the $20\times 20\;{\text{cm}}^{2}$ applicator at electron energies of (a) 4 MeV, (b) 6 MeV, (c) 9 MeV, (d) 12 MeV, and (e) 16 MeV.

Figures 4(a), (b), (c), (d), and (e) show the ROFs plotted against the radii of the circular cutouts for the $10\times 10\;{\text{cm}}^{2}$ applicator with electron energies of 4 MeV, 6 MeV, 9 MeV, 12 MeV, and 16 MeV, respectively; Figs. 5(a), (b), (c), (d), and (e) show a similar set of plots for the $20\times 20\;{\text{cm}}^{2}$ applicator. Both figures show the measured ROFs with fitting curves using the polynomial (broken line) and exponential (solid line) method as mentioned in Section II.D. Due to the limited space in this paper, similar plotting for the other three applicators is not shown. Table 3 shows a comparison of the ROFs predicted by eMUc with the measurements for specific irregular fields using various energies and applicators. Table 4 shows the percentage differences between the predicted and measured doses at different energies, applicators, interpolation methods, and SSDs.

**Table 3:** A comparison of calculated ROFs and measurements for irregular fields using different energies and applicators. All ROFs were measured at SSD=100cm. An irregular cutout the same as (Fig. 3a) was used for the $6\times 6\;{\text{cm}}^{2}$ applicator, and cutouts the same as (Fig. 3b) were used for other applicators.

Energy (MeV)	Applicator (cmxcm)	Measured ROF	Predicted ROF (Poly.)	Percentage difference (%)	Predicted ROF (Exp.)	Percentage difference (%)
4	10×10	0.934	0.930	−0.5	0.932	−0.3
4	15×15	1.003	0.990	−1.3	0.991	−1.3
6	15×15	0.997	0.987	−1.0	0.986	−1.1
6	20×20	1.014	1.003	−1.0	1.004	−0.9
9	10×10	0.896	0.897	0.1	0.897	0.1
9	15×15	0.974	0.965	0.1	0.966	−0.8
9	20×20	0.980	0.970	−1.0	0.972	−0.8
12	6×6	0.787	0.789	0.2	0.786	−0.1
12	15×15	0.964	0.955	−0.9	0.958	−0.6
12	20×20	0.972	0.961	−1.1	0.962	−0.9
16	6×6	0.894	0.899	0.6	0.901	0.8
16	10×10	0.933	0.926	−0.7	0.926	−0.7

**Table 4:** A comparison of predicted doses based on the calculated electron MUs by eMUc and measured doses for irregular fields at different energies, applicators, interpolation methods, and SSDs. Irregular fields the same as (Fig. 3b) were used for different applicator sizes.

Energy (MeV)	Applicator (cmxcm)	SSD (cm)	Measured Dose (cGy)	Predicted Dose (Poly.) (cGy)	Percentage Difference (%)	Predicted Dose (Exp.) (cGy)	Percentage Difference (%)
4	10×10	100	100.5	101.5	1.04	102.3	1.83
4	15×15	100	102.0	103.8	1.79	103.8	1.79
6	10×10	100	100.3	101.5	1.16	101.5	1.16
6	15×15	100	100.7	101.8	1.08	101.5	0.78
6	15×15	105	90.5	91.5	1.12	91.2	0.78
6	15×15	110	81.4	82.7	1.58	82.4	1.22
9	10×10	100	100.2	101	0.79	100.8	0.59
9	10×10	105	89.5	90.8	1.44	90.6	1.22
9	15×15	100	100.2	101	0.92	100.5	0.32
9	15×15	105	89.8	90.9	1.18	90.3	0.51
9	15×15	110	80.8	81.7	1.01	81.7	1.01
12	10×10	100	98.7	98.6	−0.11	98.8	0.06
12	10×10	105	88.0	88.7	0.85	88.9	1.03
12	10×10	110	79.2	80.3	1.29	80.4	1.46
12	15×15	100	100.1	100.7	0.56	99.8	−0.29
12	15×15	105	89.8	90.6	0.86	89.8	−0.03
16	10×10	100	97.6	98.4	0.78	98.6	0.99
16	10×10	105	87.3	88.6	1.50	88.8	1.70
16	10×10	110	79.1	80.2	1.35	80.3	1.55
16	15×15	100	99.7	100.7	0.96	99.9	0.11
16	15×15	105	89.5	90.7	1.32	89.9	0.42
16	15×15	110	80.8	81.4	0.69	81.35	0.69

## IV. DISCUSSION

In Fig. 4, it can be seen that both curve‐fitting methods perform well in the interpolation and extrapolation of the ROF data. The two fitting curves also match well in the clinical size range of the cutout, and only show a small deviation (about ±1mm) when the radius of the cutout is smaller than 1 cm at low electron energies such as 4 MeV and 6 MeV. The polynomial method is commonly used to fit the ROF curve.^(^ ^11^ ^,^ ^21^ ^–^ ^23^ ^)^ However, it is noted that the polynomial fitting curves usually “shift” up for large cutout radii as shown in Fig. 4; even worse, the curves “shift” down and then up in Fig. 5 for larger applicator sizes. This “shift” is due to the mathematical characteristic of Eq. (4). For small fields relative to the size of the cone, the polynomial is a fairly accurate predictor for the field ROF because the down “shift” in one region is counterbalanced by an up “shift” in the next. When the cutout size begins to approach the size of the cone, however, the polynomial method in Eq. (4) cannot perform well because the radii for some of the angular segments of the integration are larger than the largest measured circle, and extrapolation of the polynomial curve ceases to be accurate. Therefore, in this paper, the exponential curve‐fitting method with the formula as shown in Eq. (5) is suggested. It can be seen in Figs. 4 and 5 that the exponential fit is better in both the small and large cutout size regions. It also contains only three fitting coefficients compared to five or more for the polynomial method, and therefore can simplify the database and decrease the computing time. In eMUc, both methods can be selected to calculate the MU with predicted ROF differences smaller than ±0.5% in the clinical cutout size range. Moreover, an interlock was imposed in the program when the cutout radius is larger than the polynomial method can accurately predict. It is interesting to note that in theory, a zero dose (or some very small leakage dose) should be expected at zero radius in Figs. 4 and 5. That is, the fitted curves should reach the origin of the plotting. In fact, it is found that the curves ended very close to the origin but were not exactly at the point of zero dose and zero radius. This may be due to the small uncertainty in our measurement setup and the accuracy of the curve fitting. However, this would not affect the accuracy of the ROF determination from the curves in the clinical cutout range.

Table 3 shows the measured ROFs for various energy and applicator combinations using cutouts as shown in Fig. 3. An irregular cutout the same as Fig. 3(a) was used for the $6\times 6\;{\text{cm}}^{2}$ applicator, and cutouts the same as Fig. 3(b) were used for other applicators. The table also shows the predicted ROFs using the polynomial and exponential curve‐fitting methods, and the percentage differences when they were compared to the measurements. It can be seen that the percentage differences between the measurements and predictions (both using polynomial and exponential method) are less than ±2%. Similarly, many clinical measurements and predictions by eMUc were compared and their differences were also within ±2%. The differences of the predicted ROFs between both methods are small and within ±0.5%. However, the exponential fitting method is suggested when the distance between the irregular field edge and central axis is large, and the field edge extends very near to the corner of the applicator insert. In this case, the radius of the segment circle is large, and the exponential method has a better fitting and interpolation result, as shown in Figs. 4 and 5.

Table 4 shows a comparison of the predicted and measured dose using various energies, applicators, interpolation methods, and SSDs. Irregular cutouts the same as Fig. 3(b) for all the applicators were used. In the table, different combinations of electron energies, applicators, and SSDs with an irregular cutout were used to verify the predicted dose with measurements. One hundred MUs were given to an irregular cutout, and the dose at $d_{\max}$ was measured using a Farmer chamber or radiographic film when the applicator size was small. The dose corresponding to 100 MUs at $d_{\max}$ was then predicted by eMUc using the polynomial and exponential fitting methods. The percentage differences between the predicted and measured dose are shown in the table, and it can be seen that they agree with each other within ±2%. In our experience, it took about one week to prepare all the cutouts for different applicators in the commissioning, another two weeks for the ROF measurement, and one more week for the verification.

A new feature of the program is the ability to import the shape of the electron field image into the calculator. The user only needs to scan the drawing/photocopy of the irregular cutout with a length scale using a commercial optical scanner. The scanned image can be converted to any graphical format, such as JPEG, BITMAP, and TIFF. The program can read this image file directly and display it in the screen of the front‐end window as shown in Fig. 1. Once the image is loaded into the calculator, the user can define the length scale of the cutout image by first clicking the “Set 2 cm Scale” button, and then clicking two points in the image that are 2 cm apart. The user can then use the mouse to outline the shape of the cutout field and locate the central axis within the field. The user also has the option to select the number of radials to be used in the sector integration, and can zoom in and out on the field image to allow for more accurate digitization of the field. When all parameters are input into the calculator program, the MU will be calculated and displayed in the bottom right‐hand corner of the front‐end window as shown in Fig. 1.

Another feature of the calculator is the option to print out the calculated MU result as an electronic file or in a hard copy. When the “print” option is selected from the “file” menu, the user is prompted for treatment information that was not necessary for the MU calculation, such as the patient's name and ID number, the oncologist's name, and any other comments that the user wishes to include. An electronic file will then be generated in Microsoft Word with this information in addition to the calculated MU and all the related treatment parameters. This file can be printed out either as a hard copy to be kept in a binder or it can be transferred to an electronic patient record database for storage.

It is well known that for some small cutouts, there is a significant reduction in $d_{\max}$ and the percentage isodose line covering for the target. Although for the commissioning of eMUc it is possible to measure the ROF at every reduced $d_{\max}$ for small circular cutouts, the whole measurement is very time‐consuming. In addition, radiation oncologists sometimes like to prescribe the dose at only one $d_{\max}$ (i.e., measured from the $10\times 10\;{\text{cm}}^{2}$ cutout) for consistency and convenience. In this situation, it is necessary to ensure the following: (1) The dosimetrist and the radiation oncologist should understand that all the ROFs used in eMUc are measured at $d_{\max}$ for the standard $10\times 10\;{\text{cm}}^{2}$ cutout, and (2) the physicist should discuss with the radiation oncologist when the change of $d_{\max}$ and percentage isodose could potentially affect the treatment for the patient. If necessary, ROF measurements should be carried out for each particular patient, and the MU value calculated by eMUc should only be used as a reference.

For future work regarding the program, eMUc has the potential to link to a LINAC treatment delivery system such as VARiS (Varian Medical System, Palo Alto, CA) so that the calculated MU can be transferred directly for the treatment delivery. The calculator program can also link to a treatment‐planning system such as ${\text{Pinnacle}}^{3}$. When a plan for electron radiotherapy is completed in the system, all patient‐planning parameters and information can be transferred to eMUc, which acts as a QA tool for the predicted treatment MU. For future work regarding the calculation algorithm, applicator factors could be separated from the measured doses. If this has been verified for the LINAC, the model could be simplified to a single setup measurement for one applicator size. Moreover, the range of the polynomial fit could be reduced because the measured data indicated that ROFs were unity when the cutout radii were beyond 3 cm. All works are in progress.

## V. CONCLUSIONS

A new electron MU calculator program, eMUc, was developed based on the sector‐integration algorithm. It was written using Microsoft's Visual Basic.net framework and has a user‐friendly front‐end window. A new algorithm for measuring the radius of each angular segment in an irregular cutout field for the sector‐integration routine as well as an exponential curve‐fitting method for obtaining accurate interpolation or extrapolation results were suggested. The well‐known polynomial curve‐fitting method is still used as an option in the program, although such a method does not perform well when the irregular clinical cutout size is comparable to the area of the applicator insert. The calculator program has a comprehensive image import interface, so that the scanned image file of the cutout can easily be transferred to the calculator program. The user can then use the computer mouse to outline the irregular shape of the field. This saves a lot of time compared to using the film digitizer, which is not usually available in many filmless/paperless centers to digitize the cutout drawing. An electronic file can easily be generated with a record of the patient information, the calculated MU, and the treatment parameters after the calculation. This file can either be forwarded to a patient database or printed out in hard copy. Verification between the predicted and measured results is done by comparing the ROFs measured with different shapes of irregular cutouts, energies, and applicators to those predicted by the program. Moreover, many irregular clinical cutouts are used in the verification. The accuracy of the calculator program is found to be within ±2% compared to the measurements. It is concluded that such a calculator can provide a convenient electron MU calculation platform for the dosimetrists, radiotherapists, and physicists in electron radiotherapy. Future potential developments such as connecting the calculator program to the RTPS as a QA tool are in progress.

## ACKNOWLEDGMENTS

The authors would like to thank Scott Newman and Beverly Bradley for their initial effort in working with this project. All staff contributing to the measurements for the database are acknowledged in the Grand River Regional Cancer Center. The authors thank very much the editor and reviewers for their helpful suggestions, which improved the quality and presentation of this work.
